# The Role of Functional Lumen Imaging Probe (FLIP) in Addition to High‐Resolution Manometry and Timed Barium Esophagram in Treated Achalasia Patients With Persistent or Recurrent Symptoms

**DOI:** 10.1111/nmo.70281

**Published:** 2026-03-10

**Authors:** Elise M. Wessels, Niek Warringa, Gwen M. C. Masclee, Jeroen M. Schuitenmaker, Albert J. Bredenoord

**Affiliations:** ^1^ Department of Gastroenterology & Hepatology Amsterdam Gastroenterology and Metabolism, University Medical Centers Amsterdam Amsterdam the Netherlands; ^2^ Amsterdam Gastroenterology, Endocrinology & Metabolism Amsterdam the Netherlands; ^3^ Department of Gastroenterology & Hepatology Isala Hospital Zwolle the Netherlands

**Keywords:** achalasia, functional lumen imaging probe, high‐resolution manometry, predictors, timed barium esophagram, treatment outcome

## Abstract

**Introduction:**

A subgroup of treated achalasia patients has recurrent symptoms which prompt consideration of additional treatment. The aim of this study was to examine the additional yield of functional lumen imaging probe (FLIP) to high‐resolution manometry (HRM) and timed barium esophagram (TBE) in selection of patients for retreatment.

**Methods:**

This prospective observational cohort study was performed between November 2019 and October 2025 and included treated achalasia patients with recurrent or persistent symptoms who underwent FLIP in addition to HRM and TBE prior to retreatment. The primary outcome was the association between the distensibility index measured by FLIP and response to retreatment (treatment success: Eckardt score ≤ 3).

**Results:**

In total 84 patients were included (median age 50 years, 36.9% female). At a median follow‐up of 11 weeks after retreatment, 82.1% had treatment success (*N* = 69/84) and 17.9% had treatment failure (*N* = 15/84). The distensibility index at 40 mL on FLIP did not significantly differ between patients with and without treatment success (1.4 mm^2^/mmHg vs. 1.0 mm^2^/mmHg, *p* = 0.463). Almost all patients with abnormal HRM and TBE results had treatment success (*N* = 22/23, 95.7%). In cases with inconclusive HRM and TBE results, treatment success was 83.3% when FLIP results were normal (*N* = 10/12) and 74.3% when FLIP results were abnormal (*N* = 26/35).

**Conclusion:**

FLIP did not provide added value over HRM and TBE for identifying treated achalasia patients who may benefit from further treatment. The difficulty in selecting achalasia patients for retreatment highlights the complexity of this patient population and underscores the need for further research.

## Key Points

- Subsequent treatments tend to have lower efficacy in achalasia patients with persistent or recurrent symptoms following initial treatment, reflecting the challenges in managing this complex subgroup.
- Functional lumen imaging probe did not offer additional predictive value over high‐resolution manometry and timed barium esophagram in identifying which treated achalasia patients were likely to benefit from further treatment.
- Functional lumen imaging probe outcomes demonstrated a moderate to strong correlation to high‐resolution manometry, indicating the potential use as a substitute diagnostic tool.

## Graphical abstract

Functional lumen imaging probe did not offer additional yield over high‐resolution manometry and timed barium esophagram in identifying which treated achalasia patients with persistent or recurrent symptoms were likely to benefit from further treatment.

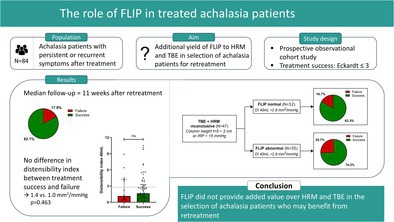

**Abbreviations**
FLIP: functional lumen imaging probe
HRM: high‐resolution manometry
IRP: integrated relaxation pressure
LES: lower esophageal sphincter
LHM: laparoscopic Heller myotomy
PD: pneumatic dilation
POEM: peroral endoscopic myotomy
TBE: timed barium esophagram

## 1 Introduction

Achalasia is a rare esophageal motility disorder characterized by absent or impaired esophageal peristalsis and incomplete relaxation of the lower esophageal sphincter (LES) [1]. This results in stasis of food in the esophagus and leads to symptoms such as dysphagia, regurgitation of undigested food, chest pain, and weight loss [2]. Achalasia is histologically characterized by degeneration of ganglion cells in the myenteric plexus, though the exact etiology remains unknown [1].

High‐resolution manometry (HRM) is the gold standard to diagnose and classify achalasia according to the fourth version of the Chicago classification [3]. Furthermore, timed barium esophagram (TBE) illustrates esophageal dilation and stasis of contrast in the esophagus and is correlated with treatment response during follow‐up [4]. The most recently developed diagnostic modality, the functional lumen imaging probe (FLIP), is a diagnostic tool which can measure the distensibility index of the esophagogastric junction by dividing the narrowest luminal cross sectional area by the esophageal pressure [5]. FLIP is increasingly performed when HRM is inconclusive or not feasible (e.g., catheter did not reach the abdomen or patient anxiety) and for tailoring achalasia treatments.

Current achalasia treatment is aimed at disrupting the LES to improve esophageal emptying and thereby reducing symptoms. The different therapeutic options include pneumatic dilation (PD), laparoscopic Heller myotomy (LHM), and peroral endoscopic myotomy (POEM) [6]. LHM and POEM are safe and effective treatment options with long‐term success rates (defined as an Eckardt score < 3 without retreatment) of 71% and 75% at 5 years follow‐up, respectively [7]. While PD also effectively reduces achalasia symptoms, it often requires repeated dilations to maintain treatment effect [8]. Despite the high efficacy rates of these interventions, a considerable proportion of patients experience persistent or recurrent symptoms after initial treatment. When these patients undergo additional treatment, the observed efficacy rates are lower compared to the efficacy rates observed in untreated achalasia patients, probably due to a lack of proper selection for retreatment [9, 10]. Recurrent symptoms may be caused by recurrent obstruction at the level of the LES, but can also result from lack of peristalsis, abnormal morphology of the esophageal anatomy, or functional symptoms. Hence, retreatment is effective only for symptom alleviation in the first situation.

In treated achalasia patients with persistent or recurrent symptoms, HRM and TBE are routinely performed to identify the cause of the recurrent symptoms and thus, to select patients for retreatment [11]. The role of FLIP in providing further insights over HRM and TBE in assessing response to retreatment has yet to be established. Therefore, the aim of this study is to examine the additional yield of FLIP to HRM and TBE in the selection of achalasia patients for retreatment.

## 2 Methods

### 2.1 Study Design

This prospective observational cohort study was performed between November 2019 and October 2025 at the Amsterdam University Medical Center. Achalasia patients with persistent or recurrent symptoms after previous treatment receive standard HRM and TBE in our center. Those who underwent FLIP in addition to HRM and TBE were asked to participate in the study. Patients who underwent retreatment after FLIP were contacted approximately 3 months after retreatment to evaluate treatment response using the Eckardt score. The decision to proceed with retreatment was guided by diagnostic test results (TBE, HRM, and FLIP) and through shared decision making with the patient, ensuring that retreatment was appropriate and aligned with patient preferences.

Eligible patients were aged 18 years or older, had a confirmed diagnosis of achalasia based on HRM and had previously undergone treatment with PD, LHM or POEM. Patients were excluded when FLIP was not successful (e.g., catheter did not reach the abdomen). Patients who did not undergo retreatment with PD, LHM or POEM after FLIP were only included in correlation analysis and in sensitivity analysis, to compare diagnostic outcomes between patients with and without retreatment. Written informed consent for reuse of patient's data was obtained from all patients prior to enrolling in the study. The Institutional Review Board concluded that formal medical ethical approval was not required for this study.

### 2.2 High‐Resolution Manometry

HRM was performed according to a standardized protocol using a solid‐state system (Manoview, Sierra Scientific, Los Angeles, USA) with 36‐channel solid state catheters with pressure sensors at 1‐cm intervals. Patients were instructed to fast at least 2 h prior to the measurement. After calibration, the catheter was transnasally placed into the esophagus beyond the esophagogastric junction with the tip of the catheter in the stomach and patients were positioned in supine position. After a baseline measurement of 30 s, participants were given 10 wet swallows of 5 mL water at 30‐s interval using a syringe [12]. Measurements were analyzed using ManoView software version 3 (Medtronic Minneapolis, Minnesota, USA). All HRM outcomes were assessed and discussed in a plenary meeting for final conclusion.

### 2.3 Timed Barium Esophagram

Patients were instructed to fast before TBE. They were positioned in an upright position and instructed to swallow 200 mL of barium contrast within 15–20 s. Radiographic images were obtained at baseline and 1, 2, and 5 min after barium contrast ingestion [13].

### 2.4 Functional Lumen Imaging Probe

FLIP 1.0 was used in this study, including an 8 cm catheter with 16 impedance sensors spaced 0.5 cm apart (EF‐325). Electrodes were positioned at both ends of the balloon and a pressure transducer was located at the distal end. The balloon was filled with saline, a conductive fluid with known electrical resistivity. When a low electric current was applied across the electrodes, voltages were measured between the impedance sensors, enabling calculation of electrical resistance, which is inversely proportional to the CSA. The distensibility index was defined as the CSA divided by the intra‐balloon pressure [14].

Calibration of the system was completed before each FLIP measurement. Patients were in an upright position during the measurement and received no sedation. The catheter was inserted transnasally and the balloon was positioned such that the EGJ aligned with the mid‐point of the balloon. After approximately 15 s, the balloon was inflated sequentially to volumes of 20–30–40–50 mL, with at least 30 s between each step. After reaching the highest volume, the balloon was deflated to 20 mL and the inflation sequence was repeated [14].

Measurements were analyzed using FLIP Analytics software (version REV13). Filter settings were configured using weighted average medium. The analytic software automatically selected multiple predefined timepoints. The position of the catheter was assessed at each timepoint and timepoints were excluded when needed, for example, due to factors such as incorrect catheter positioning or after deflation. Timepoints with an intra‐balloon pressure lower than 15 mmHg were excluded in the sensitivity analysis as low intra‐balloon pressure may overestimate the distensibility index. All FLIP measurements were independently assessed by two investigators (EMW, NW).

### 2.5 Outcome Measures

The primary outcome was the association between the distensibility index measured by FLIP and response to retreatment in previously treated achalasia patients. Secondary outcomes included the correlation between the distensibility index measured by FLIP and Eckardt scores (baseline, follow‐up and delta change in Eckardt score), as well as the correlation between the results of HRM, TBE, and FLIP.

For FLIP, the distensibility index and esophageal diameter at 30 and 40 mL were used in the analysis. Median values across multiple timepoints were used for FLIP outcomes. A cut‐off of 2.8 mm^2^/mmHg was used for the distensibility index at 40 mL. For HRM, measures of interest included integrated relaxation pressure (IRP) and baseline LES pressure. A cut‐off of 15 mmHg for IRP was applied [11]. For TBE, column height at 0, 1, 2, and 5 min was documented, along with the maximum dilation of the distal esophagus. A cut‐off value of 2 cm was applied for the column height at 5 min.

The Eckardt score was evaluated at baseline and between 1 month and 1 year after retreatment. This score consists of four questions (dysphagia, regurgitation, chest pain, weight loss) scored on a 4‐point Likert scale based on the severity of the symptom. Total scores range from 0 to 12, with higher scores indicating more severe symptoms. Treatment success was defined as a total Eckardt score ≤ 3.

Patient characteristics were extracted from medical records and included gender, age, body mass index, type of achalasia, and prior treatment. The time between last treatment before inclusion and retreatment was calculated, as well as the time between retreatment and the last moment of follow‐up.

### 2.6 Statistical Analysis

Statistical analyses were carried out using SPSS Statistics version 28.0. Continuous variables, which were non‐parametric, were reported as median with interquartile range. The Mann–Whitney U test was used to compare differences in continuous outcomes of the distensibility index between treatment success and treatment failure. Categorical variables were analyzed using either the chi‐square test or Fisher's exact test, as appropriate.

Spearman's rank correlation coefficient was used to assess correlations between the distensibility index and Eckardt scores, as well as between the results of HRM, TBE, and FLIP. A subgroup analysis was performed based on the three different retreatment options (PD, LHM, and POEM). Due to the small sample size in each subgroup, statistical tests were not performed. Sensitivity analyses were performed to assess the robustness of the findings. The first sensitivity analysis excluded timepoints where intra‐balloon pressure was below 15 mmHg during FLIP measurements [15]. In the other sensitivity analysis, patients' characteristics and results of HRM, TBE, and FLIP were compared between patients who underwent retreatment and those who did not. A two‐sided *p*‐value below 0.05 was considered statistically significant.

## 3 Results

### 3.1 Patient Selection and Characteristics

**FIGURE 1:**
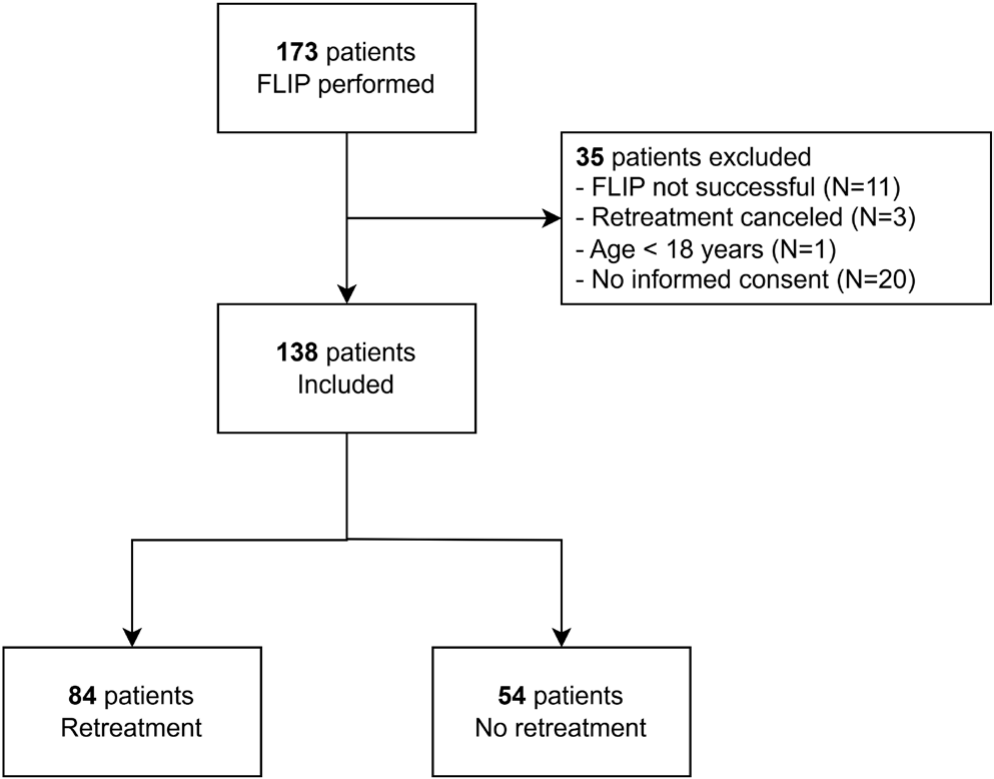
Flow chart. FLIP, functional lumen imaging probe.

In total 173 patients underwent FLIP in addition to HRM and TBE between November 2019 and July 2025. Patients were excluded for the following reasons: FLIP was not successful (*N* = 11), age below 18 years (*N* = 1), retreatment was canceled (*N* = 3), and absence of informed consent (*N* = 20). Most patients who did not respond to informed consent did not undergo retreatment. Thus, 138 patients were included in the study, of whom 84 patients underwent retreatment (Figure 1). The remaining 54 patients did not undergo retreatment and were included exclusively in correlation and sensitivity analysis.

As can be seen in Table 1, the median age of patients who underwent retreatment was 50 years and 36.9% were female (*n* = 31/84). Based on HRM at time of diagnosis, achalasia was classified as type 1 in 12 patients (*N* = 12/84, 14.3%), type 2 in 51 patients (*N* = 51/84, 60.7%) and type 3 in 9 patients (*N* = 9/84, 10.7%). Achalasia could not be classified in 12 patients (*N* = 12/84, 14.3%) as their diagnosis was based on conventional manometry performed prior to the introduction of HRM. All patients underwent at least one treatment prior to FLIP measurement and some patients (*N* = 16/84, 19.0%) received multiple treatments. Most patients underwent previous PD (*N* = 77/84, 91.7%), while 13.1% underwent LHM (*N* = 11/84) and 16.7% POEM (*N* = 14/84).

**TABLE 1:** Patient characteristics and baseline outcomes.

	All patients (*N* = 84)
*Patient characteristics*	
Age, years	50 (30)
Gender, *n* (%)	
Female	31 (36.9)
Male	53 (63.1)
Body mass index, kg/m^2^	23.9 (4.8)
Type of achalasia, *n* (%)^a^	
Type I	12 (14.3)
Type II	51 (60.7)
Type III	9 (10.7)
Not specified	12 (14.3)
Prior treatment, *n* (%)	
Pneumatic dilation	77 (91.7)
Laparoscopic Heller myotomy	11 (13.1)
Peroral endoscopic myotomy	14 (16.7)
*Baseline outcomes (before retreatment)*	
Eckardt score	5 (2)
Dysphagia	2 (1)
Regurgitation	1 (1)
Retrosternal pain	1 (2)
Weight loss	0 (1)
High‐resolution manometry^b^	
IRP, mmHg	22.0 (14.3)
Baseline LES pressure, mmHg	32.2 (15.8)
Timed barium esophagram^c^	
Column height, cm	
*t* = 0 min	7.2 (8.1)
*t* = 1 min	4.5 (6.9)
*t* = 2 min	3.3 (6.7)
*t* = 5 min	1.7 (5.3)
Max dilation, cm	3.0 (1.9)
Functional lumen imaging probe	
Distensibility index, mm^2^/mmHg	
30 mL	1.3 (2.0)
40 mL	1.5 (2.5)
Diameter, mm	
30 mL	6.4 (3.3)
40 mL	9.4 (5.1)

*Note:* Results are presented as median (IQR) unless otherwise stated.

Abbreviation: IRP, integrated relaxation pressure. LES, lower esophageal sphincter.

^a^ Based on Chicago classification version 3.0 [16].

^b^ Total *N* = 77 (treatment failure *N* = 13; treatment success *N* = 64).

^c^ Total *N* = 83 (treatment failure *N* = 15; treatment success *N* = 68).

The median Eckardt score was 5 before retreatment. HRM was successfully performed in 77 patients and demonstrated a median baseline LES pressure of 32.2 mmHg and a median IRP of 22 mmHg. TBE was successful in 83 patients, yielding a median column height of 1.7 cm after 5 min and a median maximum esophageal dilation of 3.0 cm. Median distensibility index at 30 and 40 mL was 1.3 mm^2^/mmHg and 1.5 mm^2^/mmHg respectively (Table 1).

### 3.2 Primary Outcome

**FIGURE 2:**
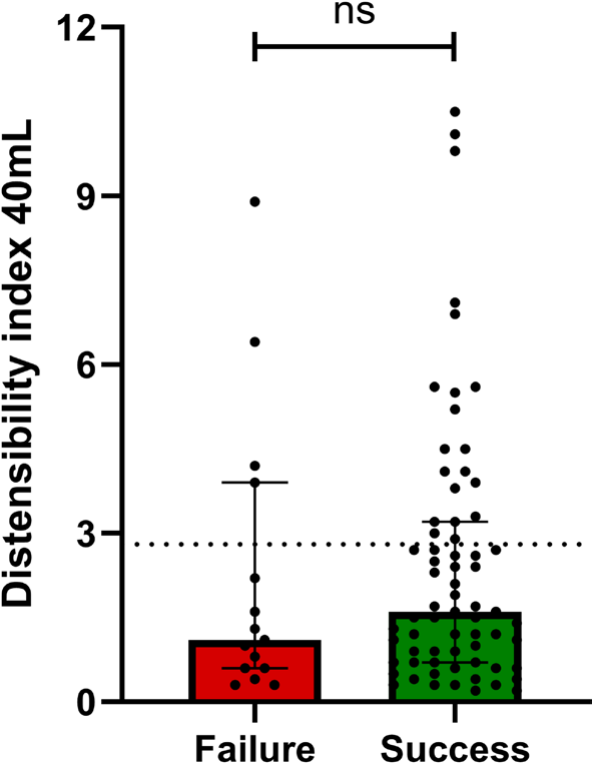
Distensibility index at 40 mL in patients with failure and success after retreatment. In total, 82.1% of the patients had treatment success and 17.9% had treatment failure. ns, not significant.

At a median follow‐up time of 11 weeks, 69 patients had treatment success (*N* = 69/84, 82.1%) and 15 had treatment failure (*N* = 15/84, 17.9%). The median time between last treatment and retreatment was 1 year. The distensibility index at 30 mL and 40 mL did not differ significantly between patients with treatment failure and treatment success (median 1.0 mm^2^/mmHg vs. 1.4 mm^2^/mmHg, *p* = 0.584 and 1.1 mm^2^/mmHg vs. 1.6 mm^2^/mmHg, *p* = 0.463 respectively) (Figure 2 and Table S1). Diameter measurements based on FLIP at 30 mL and 40 mL were also comparable between groups (failure vs. success: median 5.6 mm vs. 6.5 mm, *p* = 0.629 and 8.8 mm vs. 9.7 mm, *p* = 0.539) (Table S1).

**FIGURE 3:**
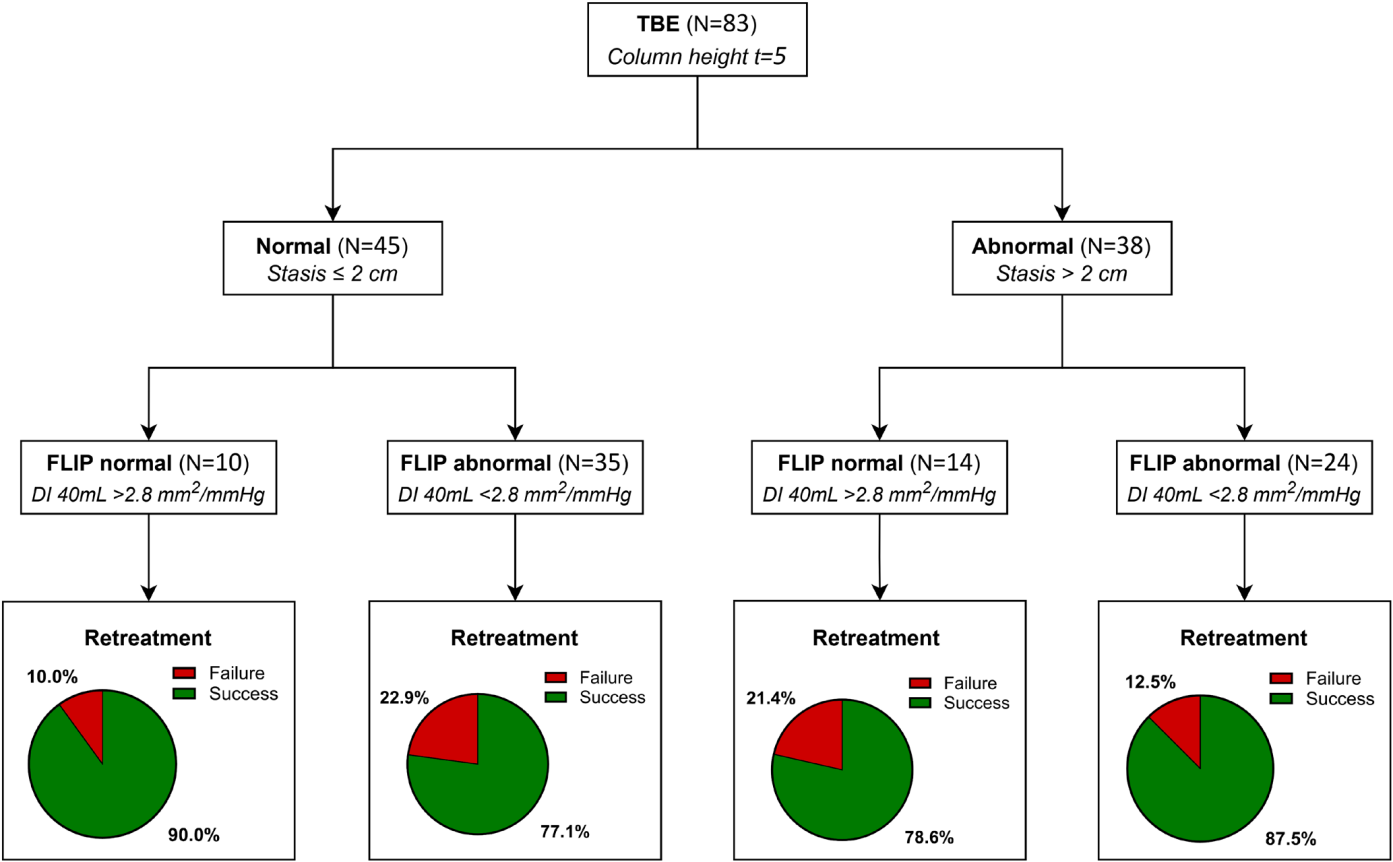
Flow chart presenting treatment success and treatment failure after retreatment, stratified by the results of timed barium esophagram (TBE) and functional lumen imaging probe (FLIP). DI, distensibility index.

**FIGURE 4:**
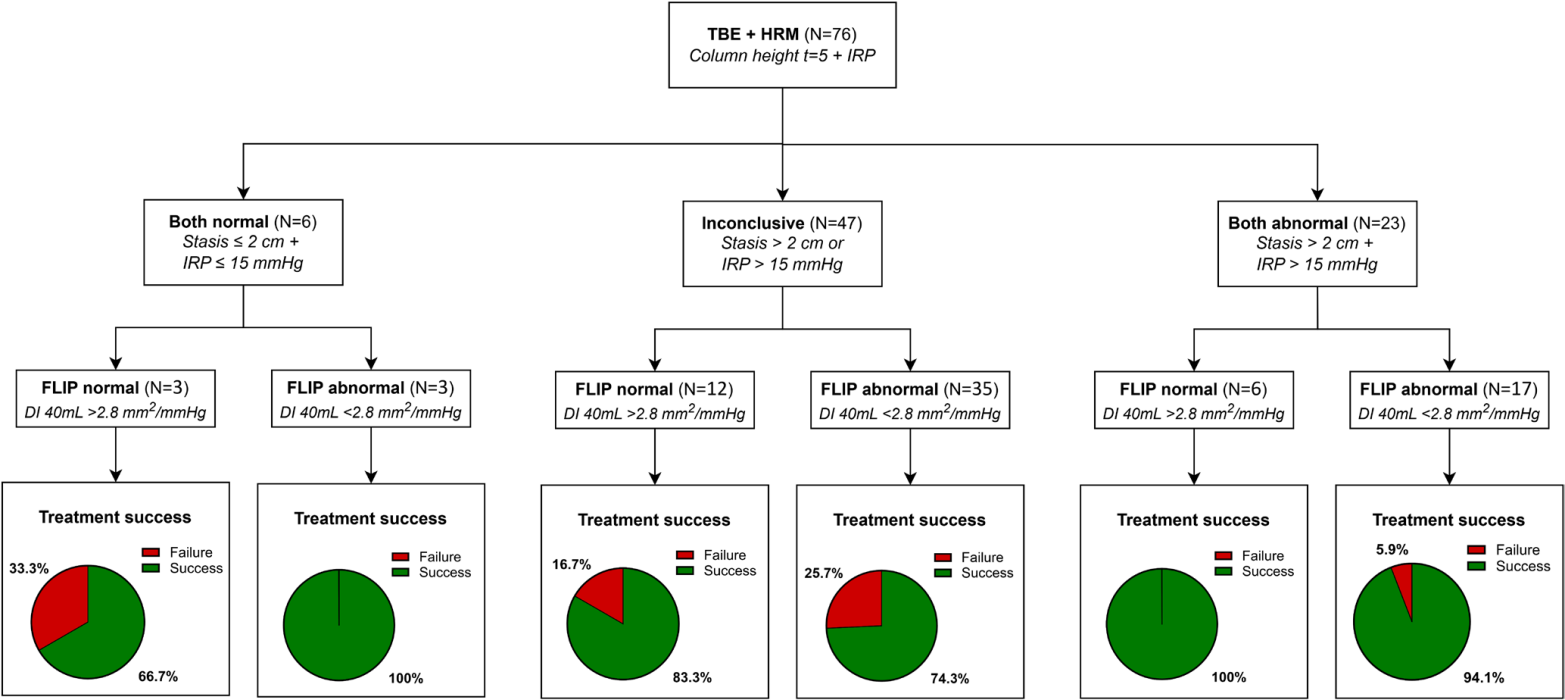
Flow chart presenting treatment success and treatment failure after retreatment, stratified by the results of timed barium esophagram (TBE), high‐resolution manometry (HRM), and functional lumen imaging probe (FLIP). DI, distensibility index. IRP, integrated relaxation pressure.

When considering HRM, TBE and FLIP as a combination score using specified cut‐off points, no significant difference was seen for the addition of abnormal tests (Table S1). In total 7.7% of the patients with treatment failure had all test results abnormal compared to 25.4% in the treatment success group. Figure 3 shows the proportion of patients with treatment success and those with treatment failure and is stratified by the results of TBE and FLIP. In patients without stasis and normal FLIP results, treatment success was 90.0% (*N* = 9/10). When stasis was observed and FLIP results were abnormal, treatment success was 87.5% (*N* = 21/24). Treatment success was 77.1% (*N* = 27/35) and 78.6% (*N* = 11/14) for inconclusive TBE and FLIP results (one result abnormal and the other normal). Figure 4 shows the proportion of patients with treatment success and failure, stratified by normal, inconclusive and abnormal outcomes of HRM and TBE and results of FLIP. Almost all patients with abnormal results of HRM and TBE had treatment success (*N* = 22/23, 95.7%). Of these 22 patients, the distensibility index at 40 mL was normal in 6 patients. In cases with inconclusive HRM and TBE results (one result abnormal and the other normal), treatment success was observed in 83.3% of those with normal FLIP results (*N* = 10/12) and in 74.3% of those with abnormal FLIP results (*N* = 26/35).

### 3.3 Secondary Outcomes

The distensibility index at 40 mL was not significantly correlated with baseline Eckardt score, Eckardt score during follow up, or the delta change in Eckardt score (Table S2). The distensibility index at 40 mL demonstrated a moderate, almost strong, inverse correlation with IRP measured by HRM (*r* = −0.591, *p* < 0.001) (Table S3 and Figure S1). Neither the distensibility index at 40 mL nor IRP correlated significantly with column height on TBE at 5 min (Table S3).

### 3.4 Subgroup Analysis

Treatment success rates were similar across different types of retreatment (Figure S2). Among patients who underwent retreatment with PD, 82.6% had treatment success (*N* = 38/46), compared to 80.8% for POEM (*N* = 21/26) and 83.3% for LHM (*N* = 10/12). Figure S3 presents the distensibility index at 40 mL stratified by type of retreatment. Statistical comparisons were not conducted due to the limited number of treatment failures in the POEM and LHM groups, which restricted analytical power.

### 3.5 Sensitivity Analysis

When comparing patients who underwent retreatment (*N* = 84) to those who did not (*N* = 54), the retreatment group had undergone POEM significantly less frequently at baseline (16.7% vs. 42.6%, *p* < 0.001). Diagnostic outcomes including IRP, baseline LES pressure and column height at 5 min were significantly higher in the retreatment group (median 22.0 mmHg vs. 12.5 mmHg, *p* < 0.001 and 32.2 mmHg vs. 18.5 mmHg, *p* = < 0.001 and 1.7 cm vs. 0.0 cm, *p* = 0.049 respectively), whereas the distensibility index at 40 mL was significantly lower compared to patients without retreatment (median 1.5 mm^2^/mmHg vs. 4.1 mm^2^/mmHg, *p* = < 0.001) (Table S4).

Excluding time points with intra‐balloon pressures below 15 mmHg did not influence the results. Although the distensibility index at 30 mL changed in 9 patients, this did not affect overall results. The median outcome at 30 mL remained consistent in the treatment failure group and changed slightly from 1.4 to 1.5 mm^2^/mmHg in the treatment success group. Furthermore, the distensibility index at 40 mL changed in only 1 patient (from 6.9 to 6.8 mm^2^/mmHg) and did not affect results in both groups.

## 4 Discussion

Achalasia treatment demonstrates high long‐term success rates for many patients; however, a subgroup of patients experience persistent or recurrent symptoms necessitating further treatment. Observed success rates after retreatment appeared to be lower compared to those after initial treatment. Therefore, appropriate patient selection is important in identifying patients who are most likely to benefit from retreatment, thereby avoiding unnecessary invasive treatments and associated complications.

Persistent or recurrent symptoms after achalasia treatment may result from various underlying causes, such as recurrent obstruction at the level of the LES impairing the passage of food to the stomach. Furthermore, an abnormal morphology of the esophageal anatomy, large epiphrenic diverticula, blow‐out myotomy, and the absence of esophageal peristalsis can mimic recurrent achalasia symptoms [17, 18, 19]. Gastroesophageal reflux after treatment, especially after POEM, may lead to regurgitation and retrosternal pain. Peptic strictures and esophageal adenocarcinoma due to longstanding reflux‐esophagitis can also present with symptoms resembling those of outflow obstruction [20]. In addition to these underlying causes, functional dysphagia and functional chest pain are commonly observed in treated achalasia patients, potentially resulting from esophageal hypersensitivity [21]. Only in patients with recurrent obstruction by a tightened LES, additional achalasia treatment may improve symptoms; yet identifying this subgroup of patients remains challenging.

Clinical guidelines recommend using TBE as initial diagnostic tool for patients presenting with persistent or recurrent symptoms [22]. However, TBE may yield inconclusive results in treated achalasia patients and in some patients, particularly in those with achalasia type 3, it can be without abnormalities. Previous studies report inconsistent findings regarding the role of TBE in predicting treatment response [23, 24]. HRM and FLIP can serve as complementary diagnostic tools, providing a more comprehensive understanding whether obstruction at the level of the EGJ is the underlying cause of recurrent symptoms. LES pressure and IRP measured by HRM have not shown to be reliable as predictors of treatment response [25, 26]. Two previous studies found that the distensibility index measured by FLIP after treatment can distinguish between patients with poor and those with adequate treatment response. Patients with poor treatment response (Eckardt ≥ 3) had significantly lower distensibility index values after treatment compared to those with adequate treatment response (40 mL: median 1.5 vs. 3.4 mm^2^/mmHg and 1.6 vs. 4.4 mm^2^/mmHg) [27, 28]. A more recent study showed no difference in the distensibility index post‐treatment between patients with treatment success and treatment failure (median 5.0 vs. 5.2 mm^2^/mmHg, *p* = 0.39), only changes in distensibility index and diameter were associated with treatment response [29].

Most studies focus on the role of HRM, TBE, and FLIP in predicting long‐term outcomes by performing diagnostics post‐treatment, rather than performing pre‐treatment diagnostics. The usefulness of performing diagnostics after either successful or failed treatment remains doubtful. It would be more appropriate to use these diagnostics prior to treatment to accurately identify patients who may benefit from treatment. In our study, we performed FLIP in achalasia patients before undergoing further treatment to assess the association between FLIP outcomes and response to the subsequent retreatment. Our results demonstrated no significant differences in the distensibility index measured by FLIP between patients with treatment success and those with treatment failure. Furthermore, nearly all patients with abnormal TBE and HRM had treatment success, suggesting no added value of FLIP in this subgroup of patients. In cases where HRM and TBE results were inconclusive or discrepant, the distensibility index measured by FLIP was not significantly different between patients with treatment success and those with treatment failure. Hence, FLIP did not provide added value when HRM and TBE were inconclusive or discrepant, nor when both HRM and TBE were abnormal.

Although FLIP did not provide added value when HRM and TBE were performed, it should not be disregarded entirely, as it may be useful for other clinical purposes. For example, FLIP performed during endoscopy could potentially substitute HRM where HRM is not available or possible. One study reported a significant correlation between the distensibility index and IRP with a correlation coefficient of −0.51 [27]. Furthermore, the distensibility index was found to outperform IRP when predicting abnormal esophageal emptying on TBE [30, 31]. Our findings confirm a significant correlation between the distensibility index and IRP with a moderate to strong correlation coefficient of −0.59. Hence, FLIP may serve as an alternative diagnostic tool in patients in whom HRM cannot be performed due to factors such as poor patient tolerance or the inability to reach the abdomen with the HRM catheter [32, 33]. Moreover, in the diagnosis of esophagogastric junction outflow obstruction, the presence of a high IRP and normal contraction pattern during HRM necessitates confirmatory testing using FLIP or TBE [3]. Some studies suggest using FLIP as an index test during upper gastrointestinal endoscopy, given that common sedatives such as midazolam, fentanyl and propofol do not significantly affect FLIP outcomes and that HRM can be omitted after FLIP in certain patients [34, 35]. Beyond its diagnostic utility, FLIP has shown some potential in tailoring treatment for achalasia patients, with higher distensibility index during treatment correlating with improved treatment response [36, 37]. However, further high‐quality controlled studies are required to strengthen clinical recommendation regarding the use of FLIP for tailoring achalasia treatment as current studies have not compared efficacy but mainly demonstrate per‐operative FLIP is feasible.

Our study assessed the additional yield of pre‐treatment FLIP to HRM and TBE in the selection of achalasia patients for retreatment, providing valuable insights for clinical decision making, helping to select patients who are most likely to benefit from retreatment. One limitation of our study is that retreatment decisions were based on the results of HRM, TBE, and FLIP, introducing a potential risk of selection bias. Comparing outcomes of HRM, TBE, and FLIP between achalasia patients who underwent retreatment and those who did not showed a significant difference in results of these diagnostic tests. This represents an important aspect in further research. Furthermore, the low rate of treatment failure may limit statistical power and increase the risk of type II error. It also did not allow us to perform multivariate regression analysis. Further research with a larger sample size would be valuable to confirm the findings of this study and to explore predictive factors for retreatment outcome. It is worth noting that we used the first version of FLIP, while the FLIP 2.0 also is capable of measuring esophageal peristalsis. However, our primary interest was in assessing the predictive value of the distensibility index of the EGJ for treatment response, which is well captured by FLIP 1.0. The follow‐up duration in this study was relatively short. The primary aim was to identify which patients can achieve treatment success in the short term after retreatment based on FLIP results in addition to TBE and HRM. However, long‐term outcomes are also important and should be addressed in further research.

In conclusion, FLIP did not provide added value over HRM and TBE for identifying achalasia patients with persistent or recurrent symptoms after prior treatment who may benefit from retreatment. The difficulty in selecting achalasia patients for retreatment highlights the complexity of this patient population and underscores the need for further research.

## Author Contributions

J.M.S. and A.J.B. designed the study. A.J.B. supervized the project. J.M.S. and E.M.W. collected the data and were responsible for project administration. E.M.W. performed the data and statistical analysis. All authors contributed to the interpretation of the results. E.M.W. wrote the manuscript with input from all authors. All authors had full access to the data and approved the final manuscript.

## Conflicts of Interest

E.M.W., G.M.C.M., and J.M.S. have nothing to declare. N.W. received speakers fee from NVMDL and has a consultancy agreement with Laborie. A.J.B. received research funding from Sanofi/Regeneron, Uniquity, Aqilion, Laborie, and Dr. Falk Pharma and received speaker and/or consulting fees from AlfaSigma, Apogee, Uniquity, Laborie, BMS, Dr. Falk Pharma, Eupraxia, Aqilion, Alimentiv, Sanofi/Regeneron, Reckitt, Domain, and AstraZeneca.

## Supporting information


**Table S1:** Difference in FLIP outcomes and combination score using specified cut‐off points in patients with treatment failure and success after retreatment.
**Table S2:** Correlation between distensibility index at 40 mL with baseline, follow‐up and delta change in Eckardt score between baseline and after retreatment.
**Table S3:** Correlation between IRP, column height at 5 min and distensibility index at 40 mL.
**Table S4:** Differences in patient characteristics and baseline outcomes of diagnostic tests between patients with and without retreatment (sensitivity analysis).
**Figure S1:** Correlation between IRP and distensibility index at 40 mL. *r* = −0.591, *p* < 0.001.
**Figure S2:** Treatment success and failure in patients undergoing retreatment, stratified by type of retreatment (subgroup analysis).
**Figure S3:** Results of distensibility index at 40 mL, stratified based on type of retreatment.

## Data Availability

The data that support the findings of this study are available from the corresponding author upon reasonable request.
