# Supplementary material for: The Role of Functional Lumen Imaging Probe (FLIP) in Addition to High‐Resolution Manometry and Timed Barium Esophagram in Treated Achalasia Patients With Persistent or Recurrent Symptoms
Source: Neurogastroenterol Motil. 2026 Mar 10;38(3):e70281. doi: 10.1111/nmo.70281 (PMC12973156; doi:10.1111/nmo.70281)
Supplement: Supplementary file 1 — Table S1: Difference in FLIP outcomes and combination score using specified cut‐off points in patients with treatment failure and success after retreatment. Table S2: Correlation between distensibility index at 40 mL with baseline, follow‐up and delta change in Eckardt score between baseline and after retreatment. Table S3: Correlation between IRP, column height at 5 min and distensibility index at 40 mL. Table S4: Differences in patient characteristics and baseline outcomes of diagnostic tests between patients with and without retreatment (sensitivity analysis). Figure S1: Correlation between IRP and distensibility index at 40 mL. r = −0.591, p < 0.001. Figure S2: Treatment success and failure in patients undergoing retreatment, stratified by type of retreatment (subgroup analysis). Figure S3: Results of distensibility index at 40 mL, stratified based on type of retreatment. [file NMO-38-e70281-s001.docx]

*Supplementary Table 1: Difference in FLIP outcomes and combination score using specified cut-off points in patients with treatment failure and success after retreatment.*

|  | **All patients** (N=84) | **Treatment failure** (N=15) | **Treatment success** (N=69) | **P-value** |
| --- | --- | --- | --- | --- |
| **Functional lumen imaging probe**  Distensibility index, mm^2^/mmHg  30 mL  40 mL  < 2.8 at 40 mL, n (%)  Diameter, mm  30 mL  40 mL | 1.3 (2.0)  1.5 (2.5)  59 (71.1)  6.4 (3.3)  9.4 (5.1) | 1.0 (2.8)  1.1 (3.3)  11 (73.3)  5.6 (3.4)  8.8 (4.9) | 1.4 (1.9)  1.6 (2.5)  48 (70.6)  6.5 (3.3)  9.7 (5.1) | 0.584  0.463  0.832  0.629  0.539 |
| **Combination score**, n (%) ^†‡^  0  1  2  3 | 3 (3.9)  15 (19.7)  41 (53.9)  17 (22.4) | 1 (7.7)  2 (15.4)  9 (69.2)  1 (7.7) | 2 (3.2)  13 (20.6)  32 (50.8)  16 (25.4) | 0.408 |

Results are presented as median (IQR) unless otherwise stated. † The combination score represents the total number of abnormal diagnostic tests including high-resolution manometry, timed barium esophagram and FLIP and is based on the specified cut-off points (integrated relaxation pressure > 15 mmHg, column height at t=5 > 2 cm and distensibility index at 40 mL <2.8 mm^2^/mmHg). ‡ Total N=77 (treatment failure N=13; treatment success N=64). FLIP, functional lumen imaging probe.

*Supplementary Table 2: Correlation between distensibility index at 40 mL with baseline, follow-up and delta change in Eckardt score between baseline and after retreatment.*

|  | **Eckardt score** | | | | | |
| --- | --- | --- | --- | --- | --- | --- |
|  | *Baseline* | | *Follow-up* | | *Δ change* | |
|  | r | p-value | r | p-value | r | p-value |
| **Distensibility index 40 mL** | -0.253 | 0.021 | 0.025 | 0.821 | 0.118 | 0.309 |

r: correlation coefficient. * Significant correlation (p<0.05).

*Supplementary Table 3: Correlation between IRP, column height at 5 minutes and distensibility index at 40 mL.*

|  | **IRP** | **Column height t=5** | **Distensibility index 40 mL** |
| --- | --- | --- | --- |
| **IRP** | --- |  |  |
| **Column height t=5** | -0.088 | --- |  |
| **Distensibility index 40 mL** | -0.591* | 0.068 | --- |

Results are presented as correlation coefficients. * Significant correlation (p<0.05). IRP, integrated relaxation pressure.

*Supplementary Table 4: Differences in patient characteristics and baseline outcomes of diagnostic tests between patients with and without retreatment (sensitivity analysis).*

|  | No retreatment (N=54) | Retreatment (N=84) | P-value |
| --- | --- | --- | --- |
| **Patient characteristics** | | | |
| Age | 54 (26) | 50 (30) | 0.908 |
| Gender  Female  Male | 29 (53.7)  25 (46.3) | 31 (36.9)  53 (63.1) | 0.052 |
| Body mass index, kg/m^2^ | 25.0 (5.2) | 23.9 (4.8) | 0.190 |
| Type of achalasia, n (%) ^†^  Type I  Type II  Type III  Not specified | 17 (31.5)  26 (48.1)  3 (5.6)  8 (14.8) | 12 (14.3)  51 (60.7)  9 (10.7)  12 (14.3) | 0.087 |
| Prior treatment, n (%)  PD  LHM  POEM | 46 (85.2)  13 (24.1)  23 (42.6) | 77 (91.7)  11 (13.1)  14 (16.7) | 0.233  0.097  <0.001 |
| **Baseline outcomes** | | | |
| Eckardt score | 4 (2) | 5 (2) | 0.027 |
| High-resolution manometry  IRP, mmHg  Baseline LES pressure, mmHg | 12.5 (7.9)  18.5 (18.2) | 22.0 (14.3)  32.2 (15.8) | <0.001  <0.001 |
| Timed barium esophagram  Column height, cm  t = 0 minutes  t = 1 minutes  t = 2 minutes  t = 5 minutes  Max dilation, cm | 6.0 (5.4)  3.6 (6.2)  2.5 (5.0)  0.0 (3.3)  3.0 (2.0) | 7.2 (8.1)  4.5 (6.9)  3.3 (6.7)  1.7 (5.3)  3.0 (1.9) | 0.023  0.060  0.072  0.049  0.998 |
| Functional lumen imaging probe  Distensibility index, mm^2^/mmHg  30 mL  40 mL  Diameter, mm  30 mL  40 mL | 3.3 (2.4)  4.1 (3.0)  8.8 (3.1)  12.4 (3.1) | 1.3 (2.0)  1.5 (2.5)  6.4 (3.3)  9.4 (5.1) | <0.001  <0.001  <0.001  <0.001 |

*Supplementary Figure 1: Correlation between IRP and distensibility index at 40mL. r=-0.591, p<0.001.*


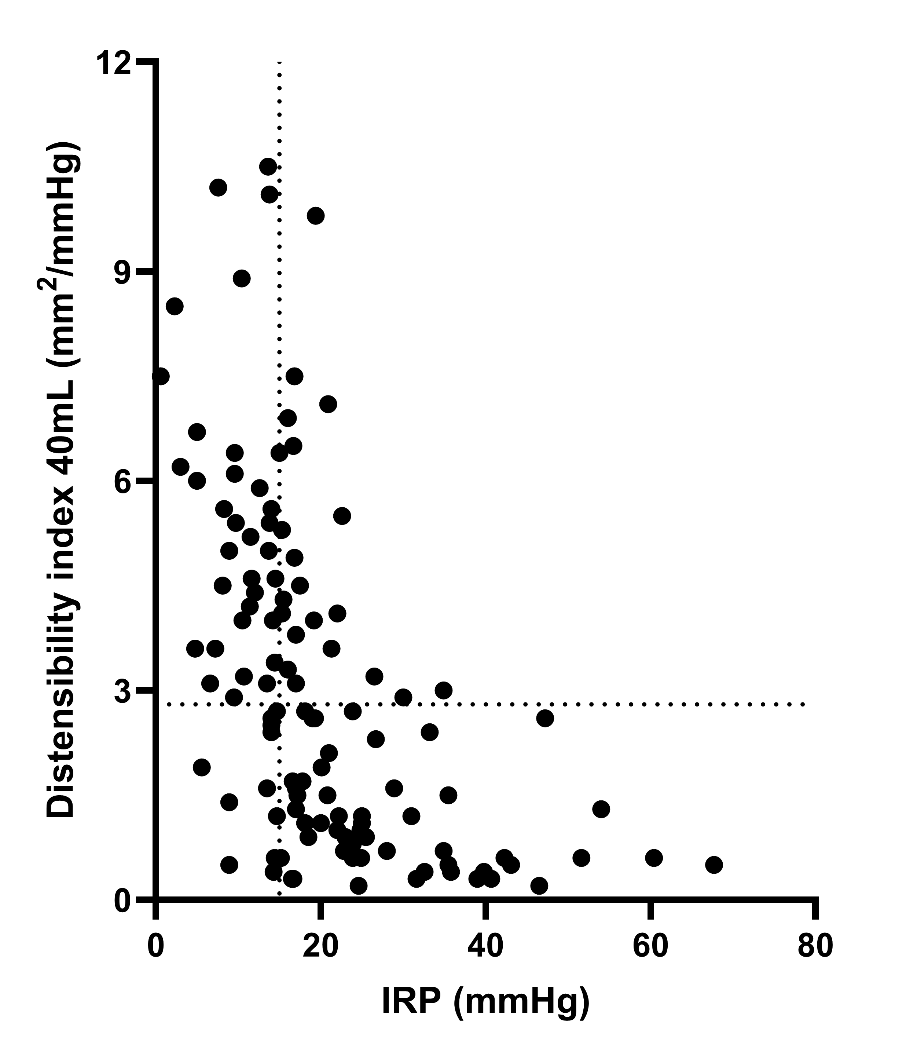


*Supplementary Figure 2: Treatment success and failure in patients undergoing retreatment, stratified by type of retreatment (subgroup analysis).*


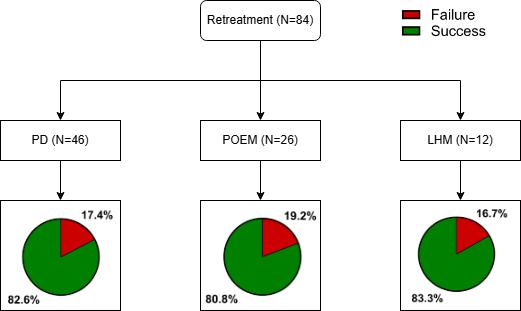


*Supplementary Figure 3: Results of distensibility index at 40mL, stratified based on type of retreatment.*

*
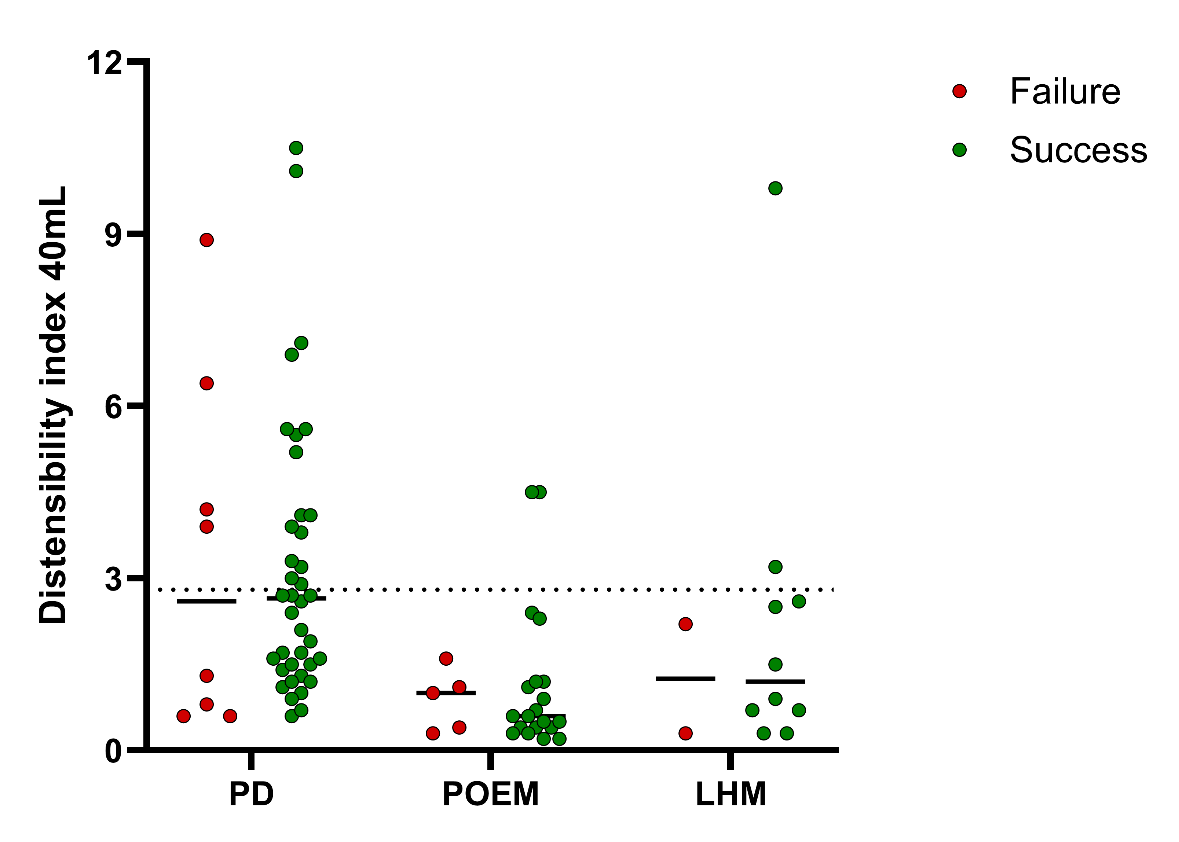
*
